# Prevalence of Malnutrition among Preschool Children in Northeast of Iran, A Result of a Population Based Study

**DOI:** 10.5539/gjhs.v5n2p208

**Published:** 2012-01-23

**Authors:** Abolfazl Payandeh, Azadeh Saki, Mohammad Safarian, Hamed Tabesh, Zahra Siadat

**Affiliations:** 1 Department of Biostatistics, School of Paramedical Sciences, Shahid Beheshti University of Medical Sciences, Tehran, Iran; 2 Department of Biostatistics and Epidemiology, School of Public Health, Ahvaz Jundishapur University of Medical Sciences, Ahvaz, Iran; 3 Department of Nutrition, Medical School, Mashhad University of Medical Sciences, Mashhad, Iran

**Keywords:** malnutrition, under weight, stunting, wasting, age specific rate

## Abstract

**Introduction:**

Malnutrition in preschool children is a significant problem and has been identified by the World Health Organization (WHO) as the most lethal form of malnutrition, indirectly or directly causes an annual death of at least 5 million children worldwide. The object of this study was to estimated the rate of underweight, stunting and wasting among preschool children in northeast of Iran.

**Methods:**

A cross sectional population based study was conducted and 70339 children; 35792 males and 34547 females were recruited. The primary outcome variables were; weight, height, age and gender of the children. The sex and age specific rate and overall rate of underweight, stunting, and wasting were calculated.

**Results:**

The rate of underweight, stunting, and wasting were 7.5%, 12.5% and 4.4% respectively. There were significant differences in stunting and wasting rate between boys and girls. The overall rate of stunting was significantly higher than the overall rates of underweight and wasting. The rate of malnutrition increased with child's age.

**Conclusion:**

In compare to WHO criteria, the rate of malnutrition among this study population was low. According to the higher rate of stunting, the main goal of future research and interventions must be finding the causes of deficiency in height growth and improving it.

## 1. Introduction

The rate of malnutrition among children is an index of development. It also represent the socio- economic status of populations. Annually 5 million children were died worldwide directly or indirectly due to malnutrition. So the World Health Organization identified that the children malnutrition is the most lethal form of malnutrition (Sarraf, Goldberg, Shahbazi, Arbuckle, & Salehi, 2005). The worldwide malnutrition estimation rates indicate that 35.8% of preschool children in developing countries are underweight, 42.7% are stunted, and 9.2% are wasted (Onis, 1993). The rate of children malnutrition in the west of Asia has been reported to be 19% (Galal, 2003). A Survey in 1995 in Iran has estimated rates of stunting, underweight and wasting nationally to be 18.9%, 15.7% and 6.6%, respectively (Ghassemi, Harrison, & Mohammad, 2002). Another research in our country has revealed that 15.4% of the children suffered from moderate to severe nutritional stunting. According to the weight for height index, 4.9% of the children had wasting. Moderate to severe underweight recorded in 10.9% of children according to weight for age index (Sayari, 2001).

These children are at high risk of mortality and morbidity, and may carry adverse health and mental consequences in their lives. Most of them live in poor societies, and with impaired physical and mental capacities.They are bound to enter a vicious cycle of poverty and malnutrition for generations to come (Alasfoor et al., 2007; World Health Organization [WHO], 1983).

The present study which uses the national survey's data conducted in 2004 in Iran, aimed to estimate malnutrition indicators in children aged under 5 years old in northeast of Iran. Also comparing these estimates with the same studies and in different age groups identified in the following.

## 2. Materials and Methods

A total of 70339 children in Khorasan province, northeast of Iran, aged 0 to 5 years were recruited in July 2004 for 20 days from those attending community clinics for routine health checks as a part of a national survey. The selected sample was 11% of study population. Anthropometric measurements were made by trained health staff using WHO methodology. Information of each subject in the sample was registered precisely. Data entry was carried out on *Epi-Info, version 6*. In order to analyze data, the resulted *Epi-Info* data file exported into the *SPSS* software, *version 11.5*.

Before doing any statistical analysis, data screening including data entry checking, missing values problems and detecting outliers performed. Detecting univariate outliers was done with computing standard scores. Scores more than 3 or less than -3 considered as outliers. Therefore a total sample of 70339 children remained.

The age groups defined in categories 0 – 5, 6 – 11, 12 – 23, 24 – 35, 36 – 47 and 48 – 59 months. The preferred method of expressing rate of malnutrition obtained through survey results is in Z-scores. The Z-scores of weight-for-age, height-for-age and weight-for-height were computed. The reference population was the NCHS/WHO. The specific rate of underweight, stunting and wasting for each age group and sex was calculated (Webb, Bhatia, & Manual, 2005). The simultaneous comparison for weight and height scores between boys and girls was test by proposed method by Tabesh et al. (Tabesh, Ayatollahi, & Towhidi, 2010).

Moderate underweight is indicated by weight-for-age Z-scores between -2 and -3, and also less than -3 considered as severe underweight. Moderate stunting was assessed as the rate of height-for-age Z-scores between -2 and -3, and also less than -3 considered as severe stunting. Also moderate wasting was assessed as the rate of weight-for-height Z-scores between -2 and -3, and less than -3 considered as severe wasting.

## 3. Results

The total sample comprised 70339 children; 35792 (50.9%) boys and 34547 (49.1%) girls. There were 1127 aged 0 – 5 months, 20230 aged 6 – 11 months, 22619 aged 12 – 23 months, 11445 aged 24 – 35 months, 7405 aged 36 – 47 months, 7513 aged 48 – 59 months. The sex specific rate and sex adjusted rate of underweight, stunting and wasting among children in the study showed in Table 1.

**Tabel 1:** Sex specific rate of underweight, stunting and wasting

Sex	% Rate of:		
	Underweight	Stunting	Wasting
**Male**	7.5	9.7	3.1
**Female**	7.6	15.4	5.8
**Overall**	7.5	12.5	4.2

**Figure 1:**
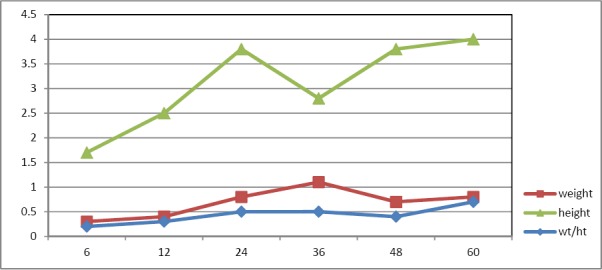
Comparing the rate of severe under weight, stunting and wasting by age

**Figure 2:**
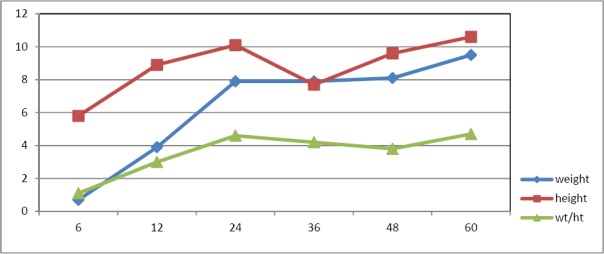
Comparing the rate of moderate under weight, stunting and wasting by age

As shown in Table 1, the sex adjusted rate of underweight among all children was 7.5% with no significant difference between boys and girls (P = 0.832). Table 2 presents the age specific rate of moderate and severe underweight in children. The rate of moderate underweight increase by age (Figure 1), but the rate of severe underweight increased till 36 months of children age and then decreased gradually (Figure 2).

**Table 2:** Age specific rate of severe and moderate underweight, and mean (SD) Z-scores of weight-for-age

Age group (months)	Severe Underweight	Moderate Underweight	Z-scores	

	%	%	Mean	SD
**0 – 5**	0.3	0.7	-2.71	0.57
**6 – 11**	0.4	3.9	-2.46	0.44
**12 – 23**	0.8	7.9	-2.46	0.42
**24 – 35**	1.1	7.9	-2.50	0.45
**36 – 47**	0.7	8.1	-2.44	0.40
**48 – 59**	0.8	9.5	-2.44	0.40
**Overall**	0.7	6.8	-2.46	0.42

The age specific rate of stunting presented in table 3. The lowest rate of stunting (7.5%) was at the age of 0 – 5 months and two peak points were observed at the ages of 48 – 59 months and 12-23 months. The Overall of stunting was 12.5% and it was significantly higher among girls (P< 0.000).

**Table 3:** Age specific rate of severe and moderate stunting, and mean (SD) height-for-age Z-scores

Age group(months)	Severe Stunting	Moderate Stunting	Z-scores	

	%	%	Mean	SD
**0 – 5**	1.7	5.8	-2.68	0.57
**6 – 11**	2.5	8.9	-2.67	0.63
**12 – 23**	3.8	10.1	-2.79	0.69
**24 – 35**	2.8	7.7	-2.71	0.70
**36 – 47**	3.3	9.6	-2.75	0.72
**48 – 59**	4.0	10.6	-2.76	0.74
**Overall**	3.2	9.3	-2.74	0.69

In order to find the major growth deficiency, the rate of underweight, stunting, and wasting separately for sever and moderate categories were compared in Figures 1 and 2. As shown in Figures 1 and 2 the rate of stunting was higher than under weight and wasting for both sever and moderate categories at all children ages (P<0.001).

Table 4 showed the age specific rate of wasting among the study population. The lowest rate of severe wasting (0.2%) was at the age of 0 – 5 months and the highest observed at the age of 48 – 59 months age group (5.4%) (Alasfoor & Mohammed, 2009). The distribution of wasting by age was bimodal, similar to stunting at ages 12-23 months and 48-59months. The Overall of wasting was 4.4% and also wasting as well as stunting was significantly higher in girls (P < 0.000).

**Table 4:** Age Specific rate of severe and moderate wasting, and mean (SD) weight-for-height Z-scores by age

Age group(months)	Severe wasting%	Moderate Wasting%	Z-scores	

			Mean	SD
**0 – 5**	0.2	1.1	-2.50	0.37
**6 – 11**	0.3	3.0	-2.47	0.38
**12 – 23**	0.5	4.6	-2.44	0.39
**24 – 35**	0.5	4.2	-2.48	0.39
**36 – 47**	0.4	3.8	-2.45	0.38
**48 – 59**	0.7	4.7	-2.49	0.45
**Overall**	0.5	4.0	-2.46	0.40
				

Once an anthropometric study has been completed, the rate of low anthropometry should be compared with other populations to assess the severity. Based on the anthropometric assessments performed in many countries around the world, proposed criteria for assessing the severity of these indications are shown in Table 5 (Gorstein et al., 1994).

**Table 5:** Proposed epidemiological criteria for assessing severity of malnutrition in populations ^a, b^

Indicator	% Rate of:			

	Low	Medium	High	Very high
**Underweight**	< 10	10.0 – 19.9	20.0 – 29.9	≥ 30
**Stunting**	< 20	20.0 – 29.9	30.0 – 39.9	≥ 40
**Wasting**	< 5	5.0 – 9.9	10.0 – 14.9	≥ 15

a. Malnutrition defined as < -2 Z-score

b. Age < 60

According to Table 5, the rate of severity of underweight, stunting, and wasting in our population, northeast of Iran, was categorized as low.

## 4. Discussion

Our findings show that all malnutrition indicators are less than 19% for our region, indicating an appropriate nutritional status in comparison to the west of Asia. In compare with research by Sayari (2001), it seems there are some better conditions in our region than the whole of Iran. Since these indicators have substantial effects on individuals, societies, and nations, they can be improved yet.

This study quantifies the magnitude of childhood malnutrition in northeast of Iran which can serve as a baseline for assessing future patterns. The prevalence of malnutrition among infants was less than other age groups it is due to high rate of breastfeeding among Iranian infants (Saki, Eshraghian, & Tabesh, 2013; Saki, Eshraghian, Mohammad, Foroushani, & Bordbar, 2010). Also an increasing pattern was observed in the rate of all three component of malnutrition by age that may due to inappropriate nutrition habits. These results made two hypotheses for future research: 1- the inappropriate nutrition among families is the cause of higher rate of malnutrition after breastfeeding cessation; 2- Attending in nursery after infancy may a risk factor for malnutrition. The present estimates can also help identify age groups and sex in need of population-wide interventions to prevent childhood malnutrition, and to encourage initiatives to establish monitoring and preventing programs (Mirmiran, Sherafat-Kazemzadeh, Jalali-Farahani, & Azizi, 2010). As indicated from our results, the rate of underweight, wasting, and stunting in girls is higher than boys, suggesting more possible attentions to them. A similar research for constructing growth curves in this area has shown that growth pattern of preschool girls is lower than boys in the same period of age (Payandeh, Tabesh, Safarian, Shakeri, & Saki, 2013).

Also the rate of stunting is higher than underweight and wasting at all ages, which implies special research, monitoring and interventions to improve the height growth (Figures 1 & 2). Complementary feeding support, early screening and management of malnutrition cases as well as social marketing to advocate and create awareness about the problem and its prevention are part of these efforts. In addition, support and collaboration from all sectors of the community is essential.
